# Access to and utilization of COVID-19 antigen rapid diagnostic tests (Ag-RDTs) among people living with HIV (PLWH): A mixed methods study from Cambodia

**DOI:** 10.1371/journal.pgph.0002940

**Published:** 2024-02-13

**Authors:** Kennarey Seang, Florian Vogt, Sovathana Ky, Vichea Ouk, John Kaldor, Andrew Vallely, Vonthanak Saphonn

**Affiliations:** 1 Grant Management Office, University of Health Sciences, Phnom Penh, Cambodia; 2 The Kirby Institute, University of New South Wales, Sydney, Australia; 3 National Centre for Epidemiology and Population Health, Australian National University, Canberra, Australia; 4 National Center for HIV/AIDS, Dermatology and STDs, Phnom Penh, Cambodia; 5 Rectorate, University of Health Sciences, Phnom Penh, Cambodia; Universidad Autonoma de Baja California, MEXICO

## Abstract

Several COVID-19 antigen rapid diagnostic tests have been approved in Cambodia, but no evidence exists about the access to and utilization of these tests. This limits public health interventions to increase testing, especially among vulnerable populations such as people living with HIV (PLWH). We conducted a mixed method study among PLWH in Phnom Penh, Cambodia, between July and August 2022 to understand their current Ag-RDT access and utilization levels, as well as key barriers and drivers. We undertook a cross-sectional survey and focus group discussions among 280 and 10 PLWH, respectively, from five HIV treatment centres using a probability-proportional-to-size and simple random sampling approach. Access was defined as having received a COVID-19 Ag-RDT within the six months and utilization as having administered a COVID-19 Ag-RDT, either to oneself or to others, within the 12 months prior to the study. We calculated means, standard deviations and proportions for continuous and categorical variables, using a linear regression model with random effects to account for clustering. Additionally, we fitted a logistic model with random effects to assess factors associated with Ag-RDT access. For the qualitative data, we used thematic analyses to identify barriers/enablers of Ag-RDT access and utilization. About 35% (n = 101) of PLWH reported having had access to an Ag-RDT test in the past six months. About 11% (n = 32) of the study participants administered the Ag-RDT to themselves, 4% (n = 10) to others and 9% (n = 24) have done both, in the past 12 months. Age and education appeared to be associated with Ag-RDT access in the logistic models. Price and advice from pharmacists were commonly reported to be the main selection criteria for the brand of Ag-RDT chosen. Ag-RDTs are an important diagnostic tool for COVID-19 among PLWH in Cambodia, but familiarity of use and price could hinder better uptake, access and utilization.

## Introduction

The first case of the coronavirus disease 2019 (COVID-19) was diagnosed in Cambodia in January 2020 [1]. Since then, a total of 137,778 confirmed cases of COVID-19 and 3,056 deaths have been reported within the country [2]. According to the latest update, 91% of total population completed two primary vaccination doses and 63% received at least one booster dose. Since July-August 2021, there has been sustained decline in transmission and no infection clusters have been detected [2].

As the pandemic escalated, it became clear that the ability to prepare for and control the pandemic depended on effective diagnostic strategies, including the availability of rapid testing [3, 4]. In Cambodia, while laboratory-based polymerase chain reaction (PCR) tests for diagnosing clinical COVID-19 infection are considered gold-standard, they are also significantly more costly and time consuming than the cheap, portable and easy-to-administer Ag-RDTs. In this context, Ag-RDTS, which can rapidly identify asymptomatic individuals with high viral load, can play a critical role in rapidly identifying and encouraging individuals with COVID-19 self-isolate, breaking chains of transmission [5]. Further, rapid diagnostics are important for efficient and early detection of COVID-19 infected individuals [6], in order to link them to care, where necessary.

Cambodia’s Ministry of Health (MoH) first approved the use of COVID-19 Ag-RDTs in Cambodia around July 2021. Several rapid antigen diagnostic test (Ag-RDTs) brands have been approved for use [7]. Following their approval, Ag-RDTs were widely used for screening purposes at ports of entry such as airports and other venues, at times free of charge and other times for a fee. Additionally, as the pandemic progressed COVID-19 testing was essential for resuming many restricted activities (e.g., international travel and economic activities) domestically and across borders [8].

PLWH are at increased risk of a severe form of COVID-19, particularly if they are of older age and present co-morbidities [9–11]. This is of particular concern in Cambodia where the latest survey among Cambodian HIV patients has revealed an average age of 45 years, with high prevalence of chronic morbidities [12]. More than 90% of the total patients on antiretroviral therapy (ART) represent an older adult population [13]. The total population of PLWH is estimated at 76,000 people [14]. Given this sizable and particularly vulnerable population of PLWH in Cambodia, access to COVID-19 testing, prevention and treatment takes on additional significance.

At the time of this research, no studies had been conducted to understand the access and utilization of COVID-19 rapid tests among the general Cambodian population, nor specifically those who are living with HIV. Such studies are of particular importance because better understanding access and utilization of Ag-RDTs among specific population cohorts can help inform optimal settings for improving rapid test uptake and access rates among those populations. In turn, improved access and utilization of COVID-19 rapid tests can potentially facilitate their timely diagnostic and linkage to treatment. Early diagnostic and timely care linkage are especially crucial for PLWH population in Cambodia (older with multiple co-morbidities). Additionally, there had been fear that the COVID-19 pandemic might slow down the progress made thus far towards the 95-95-95 target set for HIV in 2030 [15]. Effective rapid diagnostic testing strategies could be informed by the present study and help with the development of future measures to improve access to and utilization of Ag-RDTs.

Aiming to close this gap, we conducted a mixed-methods study to determine the access to and utilization of Ag-RDTs among PLWH who came to receive care at five selected HIV treatment centres in Phnom Penh, Cambodia, as well as to identify enablers and barriers to these rapid tests’ access and utilization.

## Materials and methods

### Study setting and design

In Cambodia, HIV/AIDS treatment and care clinics or facilities (called Antiretroviral Therapy (ART) sites) are under the supervision of the National Centre for HIV/AIDS, Dermatology and STD (NCHADS). We conducted a mixed method study in five ART sites in Phnom Penh, Cambodia, from July to August 2022, among PLWH who were care receivers at these ART sites. The five ART sites (out of 11 eligible ART sites in Phnom Penh) were selected using probability-proportional-to-size (PPS) sampling method, based on the national HIV database from the NCHADS (details in S1 Table). The five ART study sites received PLWH both from within Phnom Penh and also outside of this area (with about 30% of attending PLWH from the provinces).

For context, in terms of COVID-19 Ag-RDT policy, Cambodia’s Ministry of Health (MoH) first approved the use of RDT for the general public in Cambodia around July 2021. Subsequently, the availability and use of Ag-RDTs gradually increased and Ag-RDTs had become commonly available in Phnom Penh by the time this study was conducted in 2022. All five ART sites were using Ag-RDTs to screen symptomatic people coming into the care facilities, regardless of their HIV status, before allowing entry.

### Study population and data collection

The population of the qualitative study (focus group discussion) includes PLWH (n = 10), recruited purposively from the five aforementioned ART sites. The on-site study team approached the participants and invited them to join an online focus group discussion (FGD), on a voluntary basis, when they came to receive care at the selected study sites. The study team explained the procedure of the remote meeting, provided study information sheet and obtained their verbal consent. Each consented participant would then be offered a unique ID number to identify them in the study. There was a total of four group discussions (one group of four and three groups of two participants).

For the quantitative survey, a predetermined number (n = 280, based on the PPS method described earlier) of PLWH aged 18–65 years, of both sexes, who came to receive HIV care from each selected site were recruited (and consented) in a consecutive manner. Similar to the recruitment process for the qualitative study, each participant was allocated a unique identifying number for the quantitative survey. The sample size for the quantitative survey was calculated, at the alpha level = 0.05, based on the assumption that the Ag-RDT access among the general public in Cambodia was about 30%, using the formula in which precision (rather than power) is used for sample size estimation: $n=Z_{1-\propto /2}^{2}P(1-P)/d^{2}$, where *P* is the estimated proportion and *d* is the precision level (~ 5.6%). The final sample size is then calculated by multiplying *n* (257 from the above formula) with design effect (deff): 1+*ρ*(*m*−1), where *m* is the average cluster size (51 participants per cluster) and *ρ* is the intra-cluster correlation (ICC) parameter (0.001 in our study) and assumed refusal rate of ~ 3%.

Information collected from both the FGDs and the survey included demographic characteristics, access to and utilization of Ag-RDTs as well as other HIV-specific indicators, such as duration of HIV infection and ART usage. For focus group discussions, all sessions were conducted through Zoom. The arrangements for the virtual discussions were facilitated by the data team at the selected ART sites and conducted in a private setting. Study team were also present in person at each site to help with these arrangements. The survey was similarly conducted in a private space at each site, via Computer-Assisted-Person-Interview (CAPI) using ODK interface uploaded onto tablets. The data collection was conducted from July 15 to August 8, 2022. All audio recordings of the focus group discussions and the participants’ de-identified data were stored on the University of Health Sciences’ server, with access restricted to designated study data personnel. The approved study personnel could obtain the de-identified data from the server and work on it on their password-protected computer. Prior to focus group and survey administration, the data collectors underwent training on how to formulate qualitative questions, obtain verbal consent from participants, and use tablets to collect/record information from study participants for both studies.

### Ethics approval

The current study was approved by the HREA (Panel G) of the University of New South Wales (HC220035) and the National Ethics Committee for Health Research in Cambodia (NECHR #112) in March and April 2022, respectively. Each individual was given a unique ID for the study, no identifying information was collected and all study participants provided verbal consent according to the approved study protocols. Prior to the data collection, all data collectors underwent training with human-subject-certified study team. At the selected HIV clinics, due to special circumstances around PLWH (fear of stigma and discrimination), the verbal consent process was done in a private space set up at the clinic, between only the designated trained data collector and the participant (PLWH usually come to their medical visits by themselves as their HIV status might be unknown to their family members).

### Data analyses

We defined access as having received Ag-RDT for COVID-19 within the past six months of the interview date and identified the access venues. Utilization was defined as having administered the Ag-RDT either to oneself or to others within the past 12 months, regardless of the venues. In addition to capturing data on access and utilisation, we also documented the knowledge and (reported) practices of study participants regarding when and how Ag-RDTs should be used, and how they would respond hypothetically if they tested positive for COVID-19. We conducted thematic analyses for qualitative interviews using NVivo version 1.6.1 (QSR International, Burlington, MA, USA). For the survey data, means and standard deviation (SD), median and interquartile range (IQR) were calculated for continuous variables and proportions and percentages for categorical variables. We fitted a linear model in univariate analysis and reported on these proportions and percentages for each categorical variable, using mixed effects model (accounting for variability between clusters (random effects) at the ART site level), as appropriate. Similarly, for the crude and adjusted logistic mixed effects model, we modelled Ag-RDT access as a function of demographic and HIV-specific factors and reported crude and adjusted odds ratios (OR) from the mixed effects model, accounting for random clustering effect at the site level. The variables included in the adjusted model were selected based on prior knowledge and literature review [16]. All quantitative data were analysed using STATA version 17 (StataCorp LLC, College Station, TX, USA).

## Results

### Socio-demographics

We successfully collected information from 280 PLWH in the quantitative survey from the 281 we initially approached for the study (one person had refused the participation and had been later dropped from the analysis). “Table 1” presents the access and utilization data captured in the quantitative survey, disaggregated by demographic and HIV-specific characteristics. As illustrated in “Table 1”, 52% (n = 145) of the participants in the survey were female, and the mean age of the participants was 44 years old (SD, 11). More than 80% of survey participants had been diagnosed with HIV and on ART for at least two or more years. Only 25% (n = 71) of the participants had completed high school or higher-level qualifications.

**Table 1:** Selected demographics of study participants (n = 280), FIND accelerator project, 2022, Cambodia.

Demographics		Total (N = 280)		Access to Ag-RDT† (n = 101)		Utilization of Ag-RDT† (n = 66)	
		**n**	**%**	**n**	**% (95% CI)**	**n**	**% (95% CI)**
*Sex*							
	Male	135	48.2	54	39.6 (29.1, 50.1)	40	29.6 (21.9, 37.3)
	Female	145	51.8	47	32.4 (24.8, 40)	26	18.1 (10.3, 25.9)
*Age in years (mean*, *SD)*		44.1, 11.4		40.9, 12.2		39.5, 12.2	
	18–27 years	36	12.9	23	64.7 (45.7, 83.6)	16	46.5 (24.6, 68.3)
	28–37 years	32	11.4	13	40.6 (23.6, 57.7)	12	37.4 (20.3, 54.4)
	38–47 years	85	30.4	28	32.9 (22.9, 42.9)	18	20.8 (11.7, 30)
	48 years and above	127	45.4	37	29.1 (21.2, 37)	20	15.7 (9.4, 22.1)
*Education*							
	None to less than primary	104	37.1	21	20.2 (12.5, 27.9)	7	6.7 (1.9, 11.5)
	Secondary or less	105	37.5	34	30 (13.7, 46.3)	24	22.8 (14.8, 30.9)
	High school or higher	71	25.4	46	64.8 (53.7, 75.9)	35	51.4 (36.9, 66)
*Occupation*							
	Gov’t/law enforcements	28	10.0	17	60.7 (42.6, 78.8)	12	42.8 (24.5, 61.2)
	Private company/NGO	24	8.6	17	70.8 (52.6, 89)	14	58.3 (38.6, 78)
	Homemaker	44	15.7	10	22.7 (10.3, 35.1)	4	9.1 (0.6, 17.6)
	Unemployed/student	29	10.4	10	34.5 (17.1, 51.8)	7	24.1 (8.6, 39.7)
	Service/sale	134	47.9	42	31.3 (23.4, 39.2)	26	19.4 (12.7, 26.1)
	Others	21	7.5	5	23.8 (5.6, 42)	3	14.3 (0, 29.2)
*Residence*							
	Phnom Penh (capital city)	184	65.7	76	41.1 (29.5, 52.6)	53	29.5 (20.9, 37.9)
	Outside of Phnom Penh	96	34.3	25	26 (17.2, 34.8)	13	13 (0.4, 21.6)
*Time since HIV diagnosis*							
	≤ 2 years	32	11.4	16	42.3 (17.1, 67.6)	12	37 (18.7, 55.3)
	More than 2 years	248	88.6	85	33.9 (26.8, 41.1)	54	21.8 (16.6, 26.9)
*Time since ART initiation*							
	≤ 2 years	33	11.8	17	49.7 (30.6, 68.7)	13	39.4 (22.7, 56.1)
	More than 2 years	247	88.2	84	33.7 (26.6, 40.8)	53	21.4 (16.3, 26.6)

**Note:** Some categories might not add up to 100% due to rounding from mixed model

**†** Row percentages are reported here

**Fig 1:**
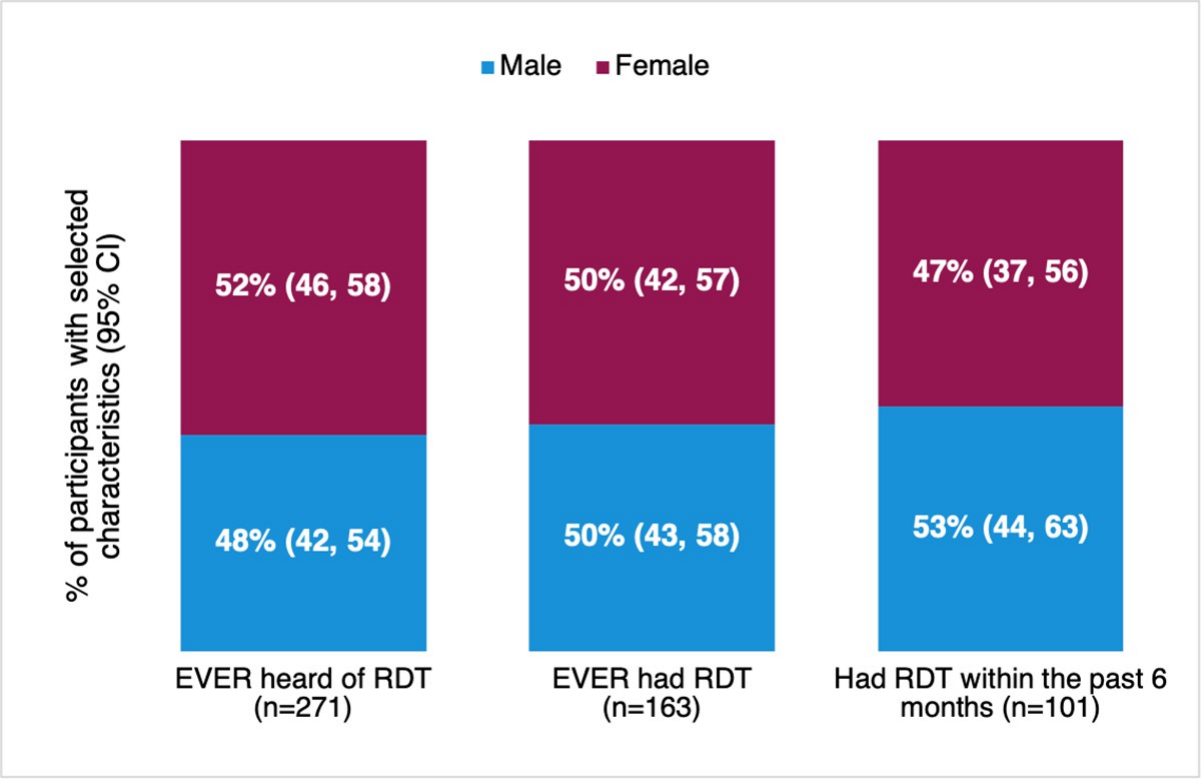
Ag-RDT access among study participants (n = 280) by sex, FIND accelerator project, 2022, Cambodia.

We found that the COVID-19 Ag-RDT access rate was similar between men and women (40% vs 32%). Similarly, the percentage of PLWH who reported having had access to Ag-RDTs in the past was also comparable between men and women (Fig 1). However, the COVID-19 Ag-RDT utilization rate was higher for men compared to women (30% vs 18%). We also observed that the PLWH in the oldest age group (48 years and above) reported the lowest rates of both Ag-RDT access and utilization (29% and 16%, respectively). This contrasted notably with those in the youngest age group (18–27 years) (65% and 46%, respectively). The highest access and utilization rates were reported by those who had completed high school or higher-level qualifications, 65% and 51%, respectively. Refer to Table 1 for more detail on access and utilization rates by demographic and HIV-specific characteristics.

### Information sources

Overall, we observed that more than 90% (n = 271) of the study participants reported having heard of COVID-19 Ag-RDTs. Approximately equal proportions of PLWH (32–35%) reported having heard of Ag-RDTs from families, friends and workplace. Online social network platforms and the media appeared to play major role in making Ag-RDTs known as 53% (n = 140) and 39% (n = 105) of the PLWH interviewed reported having heard of Ag-RDTs from an online social platform, such as Facebook, and watching television, respectively.

### Access to and utilization of Ag-RDT

Despite this high level of awareness, only 57% (n = 163) had ever undergone rapid testing, and even fewer (35% or n = 101) had undertaken rapid testing recently (within the past six months) (Table 2).

**Table 2:** Access to and utilization of COVID-19 Ag-RDT among study participants (n = 280), FIND accelerator project, 2022, Cambodia.

Characteristics			n/N	%	95% CI
**Access to COVID-19 Ag-RDT**					
*Had Ag-RDT within the past six months*					
	Yes		101/280	35.1	26.7, 43.4
	Longer than six months ago and never		179/280	64.9	56.6, 73.3
*EVER heard of Ag-RDT*					
	Yes		271/280	96.7	94.7, 98.8
	No		9/280	3.3	1.1, 5.3
*EVER underwent Ag-RDT*					
	Yes		163/280	57.4	49.8, 65.1
	No		117/280	42.6	34.9, 50.2
*Obtained Ag-RDT for free within the past 12 months*					
	Yes		70/280	25.0	19.9, 30.1
	No and never at all		210/280	75.0	69.9, 80.1
*Last Ag-RDT obtained for free from* ^*a*^					
	Public health facility		15/70	21.4	11.8, 31
	Workplace		35/70	50.0	38.3, 61.7
	Friends and families		10/70	14.3	6.1, 22.5
	Authorities/Phum/Sangkat		10/70	14.3	6.1, 22.5
*Heard (Knew) of Ag-RDT from* ^*b*^					
	Workplace		97/271	34.6	26.4, 42.8
	Friends		86/271	31.7	26.2, 37.2
	Family members		92/271	33.9	28.3, 39.6
	Social network		140/271	53.2	42.5, 63.9
	Television		105/271	39.4	32.2, 46.7
	Healthcare workers		35/271	12.9	7.3, 18.4
	Authorities/Phum/Sangkat		16/271	5.9	3.1, 8.7
*Obtaining last Ag-RDT for free was* ^*a*^					
	Easy		58/70	82.9	74, 91.7
	Hard		4/70	11.4	3.9, 18.9
	Neither hard or easy		8/70	5.7	0.2, 11.1
*Buying last RDT was* ^*c*^					
	Easy		42/52	79.0	63.2, 94.8
	Hard		5/52	10.6	0, 19.9
	Neither hard or easy		5/52	9.8	0.9, 20.3
*The cost of last Ag-RDT bought was* ^*c*^					
	Cheap		13/52	25.0	13.2, 36.8
	Expensive		16/52	30.8	16.7, 45
	Neither expensive or cheap		23/52	44.2	30.7, 57.7
*Reasons for getting last (free) Ag-RDT* ^*d*^					
	Respiratory symptoms		15/70	24.0	3.5, 44.4
	Fever		15/70	25.2	9.7, 40.7
	Exposure to known positive case		10/70	15.5	5.9, 25.2
	Exposure to suspected positive case		9/70	15.7	4.2, 27.1
	Reserve for later use		8/70	11.4	3.8, 18.9
	Required for entering events/places		28/70	40.0	28.5, 51.5
**Utilization of COVID-19 Ag-RDT**					
*Administered Ag-RDT to oneself/others within the past 12 months*					
	Yes		66/280	23.6	18.6, 28.5
		Self-administered Ag-RDT ONLY	32/280	11.4	7.7, 15.1
		Administered Ag-RDT to others ONLY	10/280	3.6	1.4, 5.7
		Self-administered Ag-RDT AND administered it to others	24/280	8.6	5.3, 11.8
	Never either way		214/280	76.4	71.4, 81.4
*If RDT (+)*, *the immediate next step should be*^*e*^					
	Seek advice from public providers		162/280	57.8	52.1, 63.6
	Seek advice from private providers		13/280	4.6	1.5, 7.6
	Seek confirmatory PCR		6/280	2.1	0.4, 3.8
	Retest with another Ag-RDT		6/280	2.1	0.4, 3.8
	Self-isolate at home		60/280	21.5	16.4, 26.5
	Seek medications from pharmacy		19/280	6.3	1.7, 10.9
*Selection of RDT depends on* ^*b*^					
	Price (cheaper, better)		111/271	40.9	34.6, 47.2
	Country of manufacturing		43/271	15.9	11.5, 20.2
	Recommendation from pharmacist		175/271	61.9	50.1, 73.8
	Test packaging		20/271	7.4	4.3, 10.5
	Nature of specimen required		16/271	7.3	2, 12.5
	Quality		31/271	11.6	7.6, 15.2

**Note:** Some categories might not add up to 100% due to rounding from mixed model

^a^ Subset to those who had obtained RDT for free within the past 12 months, single answer only

^b^ Subset to those who had ever heard of Ag-RDT, multiple answers allowed

^c^ Subset to those who had bought Ag-RDT within the past 12 months, single answer only

^d^ Subset to those who had obtained Ag-RDT for free within the past 12 months, multiple answers allowed

^e^ Single answer only

Among those who had obtained the Ag-RDT for free within the past 12 months (n = 70), 83% (n = 58) reported that free Ag-RDTs were easily available, while 11% (n = 4) said the rapid tests were hard to find. Still among them, 50% (n = 35) reported that they obtained the rapid tests from their workplace for free and 21% (n = 15) said they obtained these tests for free from public health facilities. Regarding the RDT usage, 20% (n = 15) reported that they used Ag-RDTs for symptom-based testing. Of the of the 52 PLWH who had paid for their last Ag-RDT, 79% (n = 42) reported that Ag-RDTs were readily available/easy to purchase, however only 25% (n = 13) considered Ag-RDTs to be cheap.

Regardless of where, or how (for free or paid) PLWH were accessing COVID-19 Ag-RDTs, only 23% (n = 66) among the 280 PLWH interviewed reported having utilized the Ag-RDT within the past 12 months. Breaking down Ag-RDT utilization among the 280 study participants, we observed that: 11% (n = 32) reported being familiar with self-administration of Ag-RDT, 3% (n = 10) were familiar with administering the Ag-RDT to others, and 8% (n = 24) reported being familiar with both self-testing as well as testing others.

With regard to brand selection, the majority of PLWH reported price and advice from the pharmacist to be the main selection criteria for Ag-RDTs. Finally, in terms of a follow-up step, a substantial proportion of our study participants (58% or n = 162) reported that they would next seek advice from public healthcare providers if their Ag-RDT result was positive. More details on access and utilization can be found in Table 2.

### Planned next steps in case of a positive Ag-RDT

When asked about their planned next steps if their RDT result was hypothetically positive, a large proportion of participants (n = 162 or 58%) said their next step was to seek advice from a public care provider, 21% (n = 60) said they would isolate themselves at home and about only 2% (n = 6) said that they would either seek a PCR confirmatory test or redo another RDT (Table 2).

### Factors associated with Ag-RDT access

In both the crude and adjusted mixed effect logistic models, only age, education and occupation appeared to be associated with Ag-RDT access (Table 3). Those who were in the oldest age group (48 years and above) appeared less likely to have had recent access to Ag-RDT, OR = 0.22 (95% CI 0.10, 0.48) and OR = 0.36 (95% CI 0.14, 0.94), in the crude and adjusted logistic models, respectively, compared to those in the youngest age group (18–27 years). We also observed that those who had completed high school or higher degrees were more likely to have had recent access to Ag-RDT, compared to those who had none or less than primary education, even after adjustment for sex, age, occupation, residence and time since ART initiation, aOR = 4.42 (95% CI 1.82, 10.74). In addition, we noted that sex, time since HIV diagnosis, as well as residence (whether the study participants were from the provinces and resided in Phnom Penh) were not associated with recent access to Ag-RDT. Table 4 described more details regarding Ag-RDT-access-associated factors. We did not perform the same analysis for utilization in this context of a relatively low access rate observed; it is more reasonable to examine utilization when a lot of population have access to tests but do not use them, which is not the case in our study. Furthermore, although not shown here, a brief look among those who had utilized rapid test on themselves or other showed that the majority of them had access to these tests (52/66), which confirms our assumption that low test utilization comes from low access to tests.

**Table 3:** Factors associated with Ag-RDT access among study participants (n = 280), FIND accelerator project, 2022, Cambodia.

Characteristics		Had access to RDT† (n = 101)					
		OR	95% CI	*P*	aOR‡	95% CI	*P*
*Sex*							
	Male	Ref		Ref	Ref		Ref
	Female	0.72	0.44, 1.19	0.20	1.65	0.84, 3.25	0.15
*Age*							
	18–27 years	Ref		Ref	Ref		Ref
	28–37 years	0.37	0.14, 1.02	0.05	0.52	0.16, 1.65	0.27
	38–47 years	0.27	0.12, 0.63	0.002	0.53	0.20, 1.40	0.20
	48 years and above	0.22	0.10, 0.48	< 0.001	0.36	0.14, 0.94	0.04
*Education*							
	None to less than primary	Ref		Ref	Ref		Ref
	Secondary or less	1.90	1.00, 3.60	0.05	1.74	0.87, 3.50	0.12
	Completed high school or higher	7.44	3.73, 14.8	< 0.001	4.42	1.82, 10.74	0.001
*Occupation*							
	Government/forces	Ref		Ref	Ref		Ref
	Private company/NGO	1.57	0.49, 5.02	0.45	1.09	0.28, 4.13	0.90
	Housewife	0.19	0.07, 0.54	0.002	0.23	0.06, 0.82	0.02
	Unemployed/student	0.34	0.11, 1.00	0.05	0.33	0.09, 1.17	0.09
	Service/sale	0.29	0.13, 0.68	0.005	0.34	0.12, 0.99	0.05
	Others	0.20	0.06, 0.71	0.01	0.39	0.08, 1.77	0.22
*Residence*							
	Phnom Penh (capital city)	Ref		Ref	Ref		Ref
	Outside of Phnom Penh	0.45	0.27, 0.83	0.01	0.64	0.34, 1.21	0.17
*Time since HIV diagnosis*							
	≤ 2 years	Ref		Ref	-	-	-
	More than 2 years	0.52	0.24, 1.11	0.09	-	-	-
*Time since ART initiation*							
	≤ 2 years	Ref		Ref	Ref		Ref
	More than 2 years	0.48	0.23, 1.02	0.06	0.9	0.34, 2.34	0.83

**†** Underwent Ag-RDT for COVID-19 within the past six months

**‡** Adjusted for gender, age, education, occupation and time since ART initiation

**Table 4:** Thematic analysis from patient focus group discussions (n = 10), FIND accelerator project, 2022, Cambodia.

Themes/subthemes		PLWH	
		**n/N**	**ρ**
*Sources of Ag-RDT access*			
	Workplace	7/10	0.70
	Own expenses	7/10	0.70
	Others	3/10	0.30
*Barriers to Ag-RDT utilization and access*			
	Video tutorials on test administration too fast/short	2/10	0.20
	Inadequate TV ads on how to perform test	2/10	0.20
	Misconceptions about rapid tests (nose bleeds, etc.)	4/10	0.40
	Price of tests ≥ 2 USD	10/10	1.00
*Enablers of Ag-RDT utilization and access*			
	TV ads and announcement with short videos	6/10	0.60
	Video tutorials on how to perform test	8/10	0.80
	Having someone demonstrate/explain how to use tests	8/10	0.80
*Seek advices from public hospital if tested (+)*		10/10	1
*Depression and anxiety during pandemic*		4/7	0.57

### Enablers and barriers of Ag-RDT access and utilization

We talked with 10 PLWH in the qualitative interviews. The qualitative interviews included four women and six men, with a median age of 39 years old (IQR, 18). Several important insights were also highlighted through focus group discussions. Focus group discussions were completed with 10 PLWH selected from the study sites, providing qualitative insights and understanding behind the numbers we have seen so far.

### Financial constraints

The major barrier described by all 10 of the FGD participants was the price of Ag-RDTs. One participant (PG-03) said:

*“In my mind*, *when I go to the pharmacy to buy rapid tests*, *I think if it costs more than 5–10 USD*, *I cannot afford it and will be forced to come back home (without tests)*. *Around 3 USD*, *I could maybe get them 3 or 4 times…”*. PG-03 also added that *“…regarding price*, *I’d like it to be below 2 dollars*, *not too expensive*, *our people’s earnings are still limited…”*

PG-005 shared similar view on pricing points:

“*… I think that at 1 US dollar per test*, *maybe our population could afford to get these (rapid) tests*, *otherwise*, *they will not have the ability ((to buy these tests)…”*

### Lack of clear simple tutorials videos or demonstration on test administration

The majority of the FGD participants (eight out of 10) thought that having clear tutorials videos as well as in-person demonstrations would be extremely helpful in improving their utilization. When asked about administering the Ag-RDT, one study participant (PG-09) said:

“*…should have a wide and general announcement in YouTube and social media*, *and demonstrations from the pharmacist (on how to administer rapid test) can easily help us administer the tests ourselves…”*

Similarly, another study participant (PG-10) also said:

“*…video tutorials on how to administer rapid tests in YouTube and social media*, *and demonstrations from the pharmacist can easily help us administer the tests ourselves…”*

PG-10 also added:

“*…For me*, *I just want a wider dissemination of news (on rapid tests) …those who are living in the provinces or countryside*, *they do not understand (know)”*

The same predicament also reported by another study participant (PG-02), who mentioned that despite having bought the Ag-RDT, they did not know how to use it:

“*…*.*I brought the tests but I don’t know how to use them…”*

### Misconceptions about rapid tests

Four FGD participants expressed misconceptions about COVID-19 rapid tests, resulting in them being afraid of undergoing rapid testing. PG-05 told us that:

“….*a feeling of something being stuck in the nose*, *that’s what I heard from everyone*, *most people are afraid and so I also feel afraid”*.

Another interviewee (PG-03) said:

“….*I also do not know but I wonder like other people*, *whether or not we have rapid tests that do not require nasal swab… Other people said the rapid tests (with nasal swab) can cause or worsen nasal inflammation and nose bleeds…”*

“Table 4” provides a summary thematic analysis of the FGDs, from which these quotes were derived.

## Discussion

Ag-RDTs emerged as one of the most important public health tools for responding to the COVID-19 pandemic as they are simple to use, can provide rapid results, and have been proven to detect almost all of the persons with high viral load, who are at the greatest risk of transmitting the virus [17]. Despite the fact that PLWH might be more susceptible to serious complications from COVID-19 and other pandemic-related-HIV-service disruptions [18], and that better understanding the testing access and uptake rates might be key to developing effective testing strategies and improving their overall engagement and wellbeing outcomes, studies addressing access to, utilization of and enablers/barriers to COVID-19 Ag RDT testing are very limited. At the time of writing, only a few related articles considering factors influencing healthcare access during the pandemic [19] and barriers to home isolation as a public health measure to stop the spread of COVID-19 [20] had been published, and these considered different factors than our study.

However, there are two studies, from considerably different contexts (England and the USA), which can provide some interesting comparisons. A 2022 research article examining motivations of individuals seeking COVID-19 rapid tests at two community testing centres in England reported that about 35% of their study population had heard of the rapid testing centres from their work, 8% from local authorities, 11% from Facebook and television [5]. These findings were similar to ours in terms of workplace and authorities, but quite different in terms of Facebook and television, where the figures were at 53% and 39%, respectively. It is noteworthy that the populations of both studies were very different in many aspects, and not only in terms of their HIV status. The English study population primarily included education workers, essential workers and health staff [5], whereas the majority of PLWH in our study reported sales and services as their occupation. It is, therefore, reasonable that those who sought rapid testing for COVID-19 in the English study have known about rapid testing mostly from their workplace. Additionally, social network platforms, such as Facebook and the internet are highly popular among the Cambodian population [21, 22].

Similarly, a qualitative study conducted in the US last year found that one of the most common themes reported by their interviewees was that families and friends were influencing factors of COVID-19 test uptake [23]. This is certainly consistent with both our survey and focus group discussion results. According to the Morbidity and Mortality Weekly Report (MMWR) issued in March 2021, about 11% of online survey respondents reported using an at-home rapid antigen COVID-19 test use over the past 30 days [24]. This is hard to directly compare with our finding due to the different defined timeframe and the fact that our study did not specifically refer to only at-home testing. Nevertheless, among those MMWR survey participants who did the rapid tests at home, 29% reported that they did the test because they had COVID-19 symptoms [24], and a comparable proportion of our study participants (25%) also reported symptoms as the reason for their last rapid testing.

We noticed that very few studies examined the factors associated with COVID-19 Ag-RDT access and uptake, making it difficult for us to draw comparisons in relation to other findings. One paper in particular had modelled COVID-19 testing rates by function of community-level indicators, such as unemployment rate and education; the authors concluded that these indicators were indeed associated with the COVID-19 testing rates [16]. It is hard, however, to compare their finding to ours, because their modelling was done at the group level (ecological study) and not at the individual level as ours.

In terms of barriers to testing, the most cited barriers according to a review paper published last year were the cost of testing and low health literacy [25]. These are in line with our findings from the focus group discussions, in which the PLWH interviewed reported that the price of the rapid tests had been their major concern. Several of our interviewees also reported misinformation about rapid tests, which could very well be attributed to low health literacy. However, the aforementioned review paper focused on the gold standard of COVID-19 testing, which is the molecular PCR rather than a rapid test.

Although considerable effort has been made to generate robust evidence, this study still suffers from several limitations. First, it is noteworthy that due to the sampling method selected, these numbers cannot not be appropriately presented by study sites, as some smaller sites had less than 20 study participants to contribute to the overall sample size of the entire study. However, all numbers reported here have taken into account differences across study sites (clustering effects), considering, therefore, each respective study site is a cluster. Similarly, because we were only able to recruit PLWH from five ART sites across Phnom Penh, our findings might not be suitable for generalization to those PLWH who received their HIV care at the ART sites outside of Phnom Penh. Regardless, we observed similar access rates between PLWH who were residing in Phnom Penh and those who came from the provinces to receive care at ART sites in Phnom Penh in our study, even though the proportion of PLWH from the provinces were only 30% of the total study population.

Our focus group discussions were undertaken via the unconventional method of remote meetings via Zoom, instead of the more routine face-to-face discussions. There were occasional technical challenges during some of these online sessions resulting in some interrupted video recordings, which made it hard for quality transcription. The remote discussion format also made it harder to observe the body language of the interviewees. However, during the COVID-19 pandemic, a lot of physical meetings, including medical consultations and other training sessions, at the ART sites were conducted online. The data clerks at the sites became quite familiar with these technologies and were adept at convening these remote sessions with the patients and study team. As a result, any technical difficulties during the Zoom focus group discussion sessions were minimal.

Despite these limitations, our study also presented several strengths. Our study is the first to examine COVID-19 testing access, especially one with rapid tests, among PLWH in the country. The five ART sites selected through PPS sampling method captured a good proportion of PLWH, providing a good representation of those who receive care in Phnom Penh. It should be noted as well that Phnom Penh accounted for the largest proportion of PLWH in Cambodia. Moreover, we also complemented the quantitative survey with a qualitative assessment of practices related to rapid antigen testing for COVID-19 among PLWH. This greatly added to the study, providing new insights, and a deeper understanding of the context and the numbers reported in the survey. Finally, it should be noted that we had almost no refusal in our survey so, bias from non-participation is highly unlikely.

## Conclusions

Lack of access to and utilization of simple screening tools, such as antigen testing, will certainly hinder control of transmission and ultimately of the pandemic. For the population of PLWH specifically, limited access and utilization could also negatively affect the important HIV milestones achieved globally and in Cambodia so far. Although there are limitations to the COVID-19 Ag-RDTs regarding their accuracy, their ability to screen infected individuals with high viremia and provide rapid results remain undoubtedly one of the most important public health measures in the pandemic response. While the COVID-19 affects everyone, there are undeniably population groups who are more vulnerable than others in terms of morbidity and mortality risk and for this very reason, ensuring equity in access to and uptake of rapid diagnostic tools especially among those living with HIV is crucial to enable them to more easily protect themselves and others as well as seek appropriate treatment and support in a timely manner. Multiple factors could affect test utilization ability but education on COVID-19 related symptoms and news as well as simple demonstration or instructions on correct administration of rapid tests through social media or television could be extremely beneficial even for those of low educational background and reach wider audience. While friends, families and workplace could play significant role in motivating testing uptake and promoting correct COVID-19-related education and health messages, test price and misinformation might hinder better uptake and utilization of this effective public health tool.

## Supporting information

S1 Table Distribution of study participants (n = 280) across the 5 selected ART sites according to the PPS sampling method, FIND accelerator project, 2022, Cambodia. (DOCX)

S1 File Inclusivity in global research. (DOCX)
