# Supplementary material for: Access to and utilization of COVID-19 antigen rapid diagnostic tests (Ag-RDTs) among people living with HIV (PLWH): A mixed methods study from Cambodia
Source: PLOS Glob Public Health. 2024 Feb 13;4(2):e0002940. doi: 10.1371/journal.pgph.0002940 (PMC10863891; doi:10.1371/journal.pgph.0002940)
Supplement: S1 Table — (DOCX) [file pgph.0002940.s001.docx]

**S1 Table.** **Distribution of study participants (n=280) across the 5 selected ART sites according to the PPS sampling method, FIND Accelerator Project, 2022, Cambodia.**

| **Study site** |  | **PLWH (n=280)** | |
| --- | --- | --- | --- |
|  |  | **n** | **%** |
| Clinic 1 |  | 18 | 6.4 |
| Clinic 2 |  | 31 | 11.1 |
| Clinic 3 |  | 62 | 22.1 |
| Clinic 4 |  | 81 | 28.9 |
| Clinic 5 |  | 88 | 31.4 |
| Note: the actual names of the clinics or hospitals had been removed for privacy reasons, denotations of clinic 1 to 5 are used instead. | | | |
